# Global analysis of transcription in castration-resistant prostate cancer cells uncovers active enhancers and direct androgen receptor targets

**DOI:** 10.1038/srep33510

**Published:** 2016-09-19

**Authors:** Sari Toropainen, Einari A. Niskanen, Marjo Malinen, Päivi Sutinen, Minna U. Kaikkonen, Jorma J. Palvimo

**Affiliations:** 1 Institute of Biomedicine, University of Eastern Finland, Kuopio, Finland; 2 A.I. Virtanen Institute, University of Eastern Finland, Kuopio, Finland

## Abstract

Androgen receptor (AR) is a male sex steroid-activated transcription factor (TF) that plays a critical role in prostate cancers, including castration-resistant prostate cancers (CRPC) that typically express amplified levels of the AR. CRPC-derived VCaP cells display an excessive number of chromatin AR-binding sites (ARBs) most of which localize to distal inter- or intragenic regions. Here, we analyzed direct transcription programs of the AR in VCaP cells using global nuclear run-on sequencing (GRO-seq) and integrated the GRO-seq data with the ARB and VCaP cell-specific TF-binding data. Androgen immediately activated transcription of hundreds of protein-coding genes, including IGF-1 receptor and EGF receptor. Androgen also simultaneously repressed transcription of a large number of genes, including MYC. As functional enhancers have been postulated to produce enhancer-templated non-coding RNAs (eRNAs), we also analyzed the eRNAs, which revealed that only a fraction of the ARBs reside at functional enhancers. Activation of these enhancers was most pronounced at the sites that also bound PIAS1, ERG and HDAC3, whereas binding of HDAC3 and PIAS1 decreased at androgen-repressed enhancers. In summary, our genome-wide data of androgen-regulated enhancers and primary target genes provide new insights how the AR can directly regulate cellular growth and control signaling pathways in CPRC cells.

The action of androgens is essential for the development of normal prostate gland, but also the growth and progression of the prostate cancer (PCa) are androgen-dependent processes^1^. However, for still unclear reasons, androgen deprivation therapy often fails and PCa progresses to castration-resistant prostate cancer (CRPC). Although the CRPC is apparently unresponsive to androgen deprivation, possibly due to several perturbations in the androgen signaling pathway, the signaling pathway is persistently activated in CRPC cells^2^,^3^,^4^,^5^. CRPCs often overexpress androgen receptor (AR), which seems to sensitize them to various growth-stimulating effects of the androgens. Therefore, the AR and its interacting-proteins, coregulators, remain attractive targets for treating CRPC.

The AR is a testosterone and 5α-dihydrotestosterone-activated transcription factor (TF). In addition to the receptor, the receptor’s cognate DNA-binding sites (AREs), RNA polymerase II (Pol II) and general transcription machinery, transcriptional regulation by the AR requires several coregulator proteins, coactivators and corepressors^6^,^7^,^8^. Interestingly, some coregulators, such as protein inhibitor of activated STAT1 (PIAS1), can act either as a coactivator or a corepressor, depending on the AR target gene^9^. Moreover, established corepressors, histone deacetylases HDAC1, -2 and -3 have been recently shown to co-occupy chromatin also with agonist-bound AR^10^. Pioneer factors forkhead box A1 (FOXA1) and GATA-binding protein 2 (GATA2) in turn seem to prime the chromatin to allow AR to bind DNA and therefore have an important role in guiding AR onto specific chromatin loci^11^,^12^. Also other sequence-specific TFs, such as ETS-related gene (ERG) and homeobox protein HOXB13, are enriched at the AR-binding chromatin regions^9^,^13^,^14^.

Genome-wide analyses have revealed that androgen-bound AR is mostly occupying distal AR chromatin-binding sites (ARBs) at inter- and intragenic genomic regions, rather than promoter regions^9^,^11^,^15^,^16^,^17^,^18^. Distal regulatory regions of the genes, enhancers, are typically co-occupied and regulated by multiple TFs and reported to form loops with promoter regions^19^,^20^,^21^,^22^. Recent global nuclear run on sequencing (GRO-seq) data have revealed that active enhancers enriched with H3 that is mono- or di-methylated at lysine 4 (H3K4me1/2) and H3 that is acetylated at lysine 27 (H3K27ac) are often actively transcribed by Pol II, producing enhancer-templated non-coding RNAs (eRNAs)^23^,^24^,^25^,^26^,^27^. The eRNA levels correlate positively with the transcription of nearby genes and can be used to evaluate enhancer activity^23^,^26^. The eRNAs have been suggested to promote transcription by modifying the chromatin state to facilitate and enhance TF binding onto the gene regulatory elements^28^,^29^.

Since there is scarce information of the links between eRNA-producing enhancers, ARBs and androgen-induced immediate transcription programs in CRPC cells, we have in this work used GRO-seq to elucidate the early transcription programs and enhancers in CRPC-derived VCaP (Vertebral-Cancer of Prostate) cells. Our GRO-seq analyses reveal novel early responses in the transcription of genes of growth factor signaling and cancer-related pathways. In addition, we show that only a fraction of the distal ARBs is active as determined by eRNA production. Overall, our integrated GRO-seq and VCaP-cell specific TF ChIP-seq data analysis provides novel insights into the molecular mechanism of androgen signaling from distal enhancers in CRPC cells.

## Results

### Immediate transcription responses to androgen in VCaP cells

**Figure 1:**
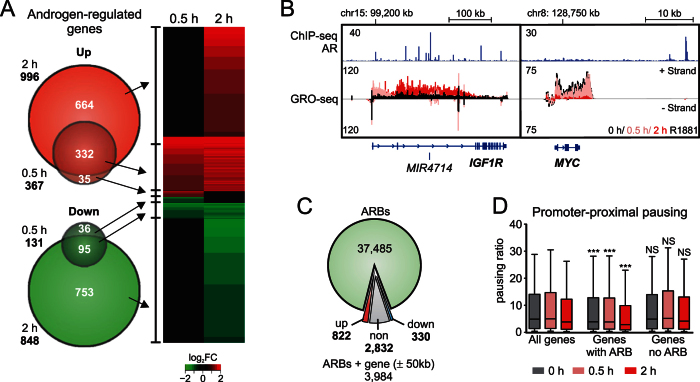
Early phase AR-regulated gene transcription in VCaP cells. VCaP cells were treated with androgen (R1881, 10 nM, 0.5 h or 2 h) or vehicle (2 h) and subjected to GRO-seq assay. The transcribed genes with RPKM ≥ 0.5, base 2 logarithmic fold change (log_2_FC) ≥ 0.585 or ≤−0.585, and FDR ≤ 0.01 were considered androgen-regulated genes. (**A**) Venn diagram (left) showing androgen-regulated genes and heat map (right) showing the changes in transcription of these genes. The color key indicates the log_2_FC of gene transcripts: up-regulated (red) or down-regulated (green) by androgen or not regulated by androgen (black). (**B**) Androgen-induced *IGF1R* locus and androgen-repressed *MYC* gene locus displayed with AR ChIP-seq (blue) and GRO-seq minus- and plus-strand signals in vehicle (black) and androgen treatment (0.5 h pink and 2 h red). (**C**) ARB association with transcribed genes. ARBs at 2-h androgen time point were associated with the closest gene TSS within 50 kb. Fractions of ARBs associated with up-, non- and down-regulated genes are marked with red, grey and blue, respectively. (**D**) Correlation of promoter-proximal pausing index (PPI) and ARBs. Box blot showing PPIs of all transcribed genes (all genes), transcribed genes with ARB (2 h) within ±50 kb of TSS (genes with ARB), and transcribed genes without ARB (2 h) association (genes no ARB). PPIs of genes with ARB association, but not those of genes without AR association, differed significantly from PPIs of all genes in respective androgen treatment time point. Significance was calculated using one-way ANOVA Kruskal-Wallis test and Dunn´s post-test (***p < 0.001). Whiskers mark 10% and 90% boundaries of the data and the line denotes median.

In order to study the direct influence of androgen on the transcription program of VCaP PCa cells, we performed GRO-seq analysis at short androgen exposure times. The analysis revealed that 498 genes were differentially transcribed (reads per kilobase per million mapped reads [RPKM] ≥ 0.5, FDR ≤ 0.01, log_2_FC ≥ 0.585 or log_2_FC ≤ −0.585) already 0.5 h after androgen exposure, and the number of differentially transcribed protein coding genes increased by 4-fold at 2 h after androgen stimulation (Fig. 1A, the genes are listed in Supplementary File 1). At 0.5 h time point, the androgen-induced genes outnumbered the repressed genes by ~3-fold, whereas at 2 h after androgen addition, the number of androgen-repressed genes was comparable to that of androgen-induced genes (Fig. 1A). This suggests that the temporal mode of action of AR on androgen-induced and androgen-repressed genes differs. Figure 1B shows *IGF1R* and *MYC* loci as examples of directly and rapidly androgen-induced and androgen-repressed genes, respectively, in VCaP cells. Both genes have been reported to be androgen-regulated in castration-sensitive LNCaP prostate cells^15^,^30^. In VCaP cells, *IGF1R* was instead recently suggested to be regulated by ERG^31^. However, based on our unbiased ChIP-seq and GRO-seq data, the *IGF1R* is a direct and immediate AR target in VCaP cells.

Association of the all AR chromatin-binding sites (AR cistrome) in VCaP cells^9^ with the 2-h AR-regulated transcriptome showed that only 3% of the ARBs reside within 50 kb of androgen-regulated genes in VCaP cells (Fig. 1C). On the other hand, 83% of androgen-induced and 39% of androgen-repressed genes associated with at least one ARB. Pol II promoter-proximal pausing is an important transcription regulation mechanism at many mammalian genes^32^. Interestingly, the promoter-proximal pausing indexes of genes associated with an ARB were generally lower than of the ones without an ARB, suggesting that the AR target genes are not generally regulated by the promoter pausing (Fig. 1D). This is exemplified by the *IGF1R* that shows no transcription at its promoter before androgen treatment (Fig. 1B).

### IGF-1 and EGF signaling are enhanced by androgen in VCaP cells

**Figure 2:**
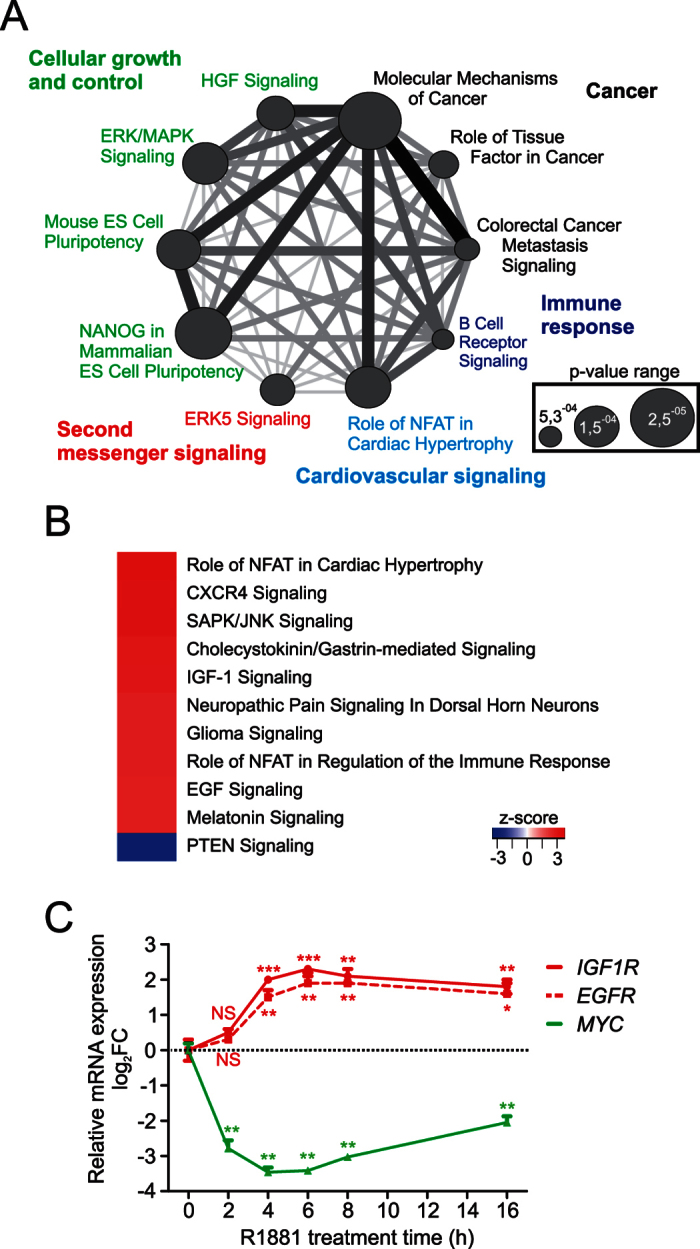
Direct AR-regulated genes enrich to cellular growth and control signaling pathways in VCaP cells. (**A**) Top ten canonical pathways enriched among AR-regulated genes (R1881, 2 h) are shown and color-coded for respective cellular function. The thickness of the connecting lines depicts the number of common genes between the pathways. The circle diameter illustrates statistical significance of the enrichment. (**B**) AR is predicted to enhance the activity of IGF-1 and EGF signaling pathways in VCaP cells. Heat map showing canonical pathways predicted to be activated (z-score ≥2, red) or attenuated (z-score ≤−2, blue) in response to androgen treatment (Fisher’s Exact Test p-value < 0.05). All listed pathways have significant z-scores (z-score ≤−2 or ≥2). (**C**) Time course of *IGF1R*, *EGFR* and *MYC* expression after addition of androgen in VCaP cells as measured using RT-qPCR and normalized with *GAPDH* mRNA levels. Fold changes were calculated with reference to 2-h vehicle sample. Data points indicate the mean of at least three biological replicates ± SDs. Student’s t-test was used to determine the significance of fold change differences between vehicle and androgen treatment (***p < 0.001, **p < 0.01 and *p < 0.05).

We next analyzed enrichment of androgen-regulated genes to canonical signaling pathways using Ingenuity Pathway Analysis (IPA). The top ten canonical signaling pathways based on the significance of the enrichment (p-value) are shown in Fig. 2A. They encompass three cancer-related pathways (e.g. molecular mechanisms of cancer, p-value 2,52E-05) and four cellular growth and control-linked pathways (e.g. ERK/MAP signaling, p-value 1,23E-04). To predict the activity of the pathways, we used the pathway activity score by IPA (z-score ≥2 or ≤−2 and Fisher’s Exact Test p-value < 0.05). Interestingly, the cellular growth pathways of IGF-1 (p-value 5,54E-04) and EGF (p-value 8,18E-03) signaling were among the signaling pathways predicted to be activated at 2 h after androgen induction (Fig. 2B). Time course experiments with RT-qPCR analyses confirmed that the expression of *IGF1R* (IGF-1 receptor gene) and that of *EGFR* (EGF receptor gene) were androgen induced, reaching maximal levels at 6 h after androgen exposure. During the same time course, *MYC* was strongly repressed by androgen (Fig. 2C).

Comparison of GRO-seq data from VCaP cells exposed to androgen for 4 h to those from LNCaP cells at the same time point revealed ≥7-fold more androgen-regulated transcripts in the VCaP cells (Supplementary Fig. S1A). Based on IPA, there were interesting differences in androgen-regulated signaling pathways between the two prostate cancer cell models: the cellular growth and control pathways in Fig. 2A were not significantly up-regulated by androgen in LNCaP cells (Supplementary Fig. S1B,C). These differences in the androgen-regulated gene programs may reflect the amplified AR levels in the VCaP CRPC cells.

### Correlation of AR chromatin binding with enhancer transcription

**Figure 3:**
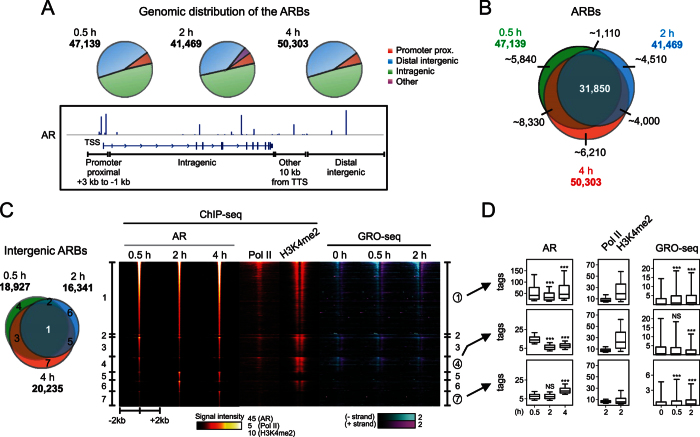
Correlation of AR chromatin binding with enhancer transcription in VCaP cells. (**A**) Genomic distribution of the ARBs in 0.5-h, 2-h and 4-h androgen-treated (R1881) VCaP cells in different genomic locations (upper panel). A schematic picture showing the genomic categories of ARBs (lower panel). (**B**) Venn diagram showing overlap of all ARBs and (**C**) intergenic ARBs (left) at indicated androgen treatment time points. (**C**) Heat map (right) with ±2 kb window centered at ARBs (R1881, 0.5 h) showing ChIP-seq signals of AR, RNA polymerase II (Pol II; R1881, 2 h), H3K4me2 (R1881, 2 h) and GRO-seq signals of vehicle- or androgen-treated (R1881, 0.5 h, 2 h) cells. Numbers on the sides of the heat map represent the overlap categories of the intergenic ARBs shown in the Venn diagram on the right. (**D**) Correlation of ARBs and GRO-seq signals. Box blot showing ChIP-seq signals of AR (with ±200 bp window), Pol II, H3K4me2 and GRO-seq signal with ±2 kb window from intergenic ARBs shared at 0.5, 2 and 4 h time points (category 1), ARBs occurring only at 0.5 h (category 4) or at 4 h (category 7). ANOVA Kruskal-Wallis test and Dunn´s post-test were used to determine the significance between 0.5- and 2- or 4-h androgen-treatment (AR tags) and between vehicle and androgen treatments (GRO-seq tags) (***p < 0.001). Whiskers mark 10% and 90% boundaries of the data and the line denotes median.

In VCaP cells, AR binds to a massive number of chromatin sites that mostly localize to inter- and intragenic regions^9^,^11^,^18^,^33^ (Fig. 3A). To investigate how the AR chromatin binding coincides with activation of enhancers and AR target gene transcription, we performed ChIP-seq time course analysis in VCaP cells exposed to androgen for 0.5 h or 4 h and included our previous at 2-h androgen-induced AR cistrome to the analysis^9^. Comparison of the cistromes at different time points, revealed that already after 0.5-h androgen exposure, the AR is occupying ~47,000 ARBs of which ~70% (~32,000) are bound by the AR also at later time points (Fig. 3B). This indicates that the holo-AR rapidly recognizes the majority of its chromatin-binding sites which remain steady during the first hours of androgen exposure. To analyze if AR chromatin-binding profile is shared between prostate cancer cell models and clinical PCa samples, we compared VCaP ARB data with those from LNCaP, LNCaP/C4-2B and 22RV1 cells and from patient samples^11^,^15^,^17^,^18^. Hierarchical clustering grouped ARBs from all cell lines together (Supplementary Fig. S2), indicating that the AR chromatin-binding profile is very similar in prostate cancer cell models. For example, the co-occurrence between our VCaP cell data and others’ LNCaP cell data^17^ was ~90%. As TMPRSS2-ERG gene fusion is present in VCaP cells^13^ but not in other cell models in this comparison, our analysis indicates that the ERG expression does not modulate the AR chromatin occupancy to a major extent. Interestingly, AR chromatin-binding pattern of some CRPC clinical samples also resembled that of the cell models, whereas such a resemblance was not seen with samples from untreated or treatment-responding patients, as noted recently^18^ (Supplementary Fig. S2).

Because the ARBs vastly outnumber the active genes, we speculated that a marked portion of the ARBs is not functional. To study the functional activity of ARBs, we integrated the GRO-seq data with the ARB data. We also analyzed the presence of active enhancer histone mark H3K4me2^27^ and Pol II from the ARBs. Even though the H3K4me2 was present at the vicinity of most ARBs, only a portion of them showed enrichment of GRO-seq signal (Fig. 3C), suggesting that all ARBs do not reside at transcriptionally active enhancers. However, the ARBs showing GRO-seq signal also displayed the presence of Pol II and enrichment of H3K4me2. Based on the heatmap (Fig. 3C), the strongest induction of the transcription by androgen seemed to be associated with the strongest ARBs, which was confirmed by a more detailed analysis of transcription changes at ARBs (Supplementary Fig. S3). To investigate the correlation of AR binding and transcription at putative enhancers in a time-dependent manner, we analyzed the changes in AR ChIP-seq and GRO-seq signals at different ARB populations (Fig. 3C,D). Intergenic sites that were occupied by the AR at all time points (group 1 in Fig. 3D) showed androgen-induced transcription, whereas the opposite was observed among the AR peaks that were unique to 0.5-h androgen exposure (group 4). The ARBs that appeared at 4 h showed weak androgen-induced transcription but with negligible H3K4me2 signal (group 7). Of note, the AR signal intensity in the group 4 and 7 was generally weaker than that in the group 1. These data suggest that the androgen-induced enhancers reside among the early-occupied and strongest AR-binding chromatin sites.

### Changes in eRNA transcription indicate that only a fraction of ARBs reside at androgen-responsive enhancers

**Figure 4:**
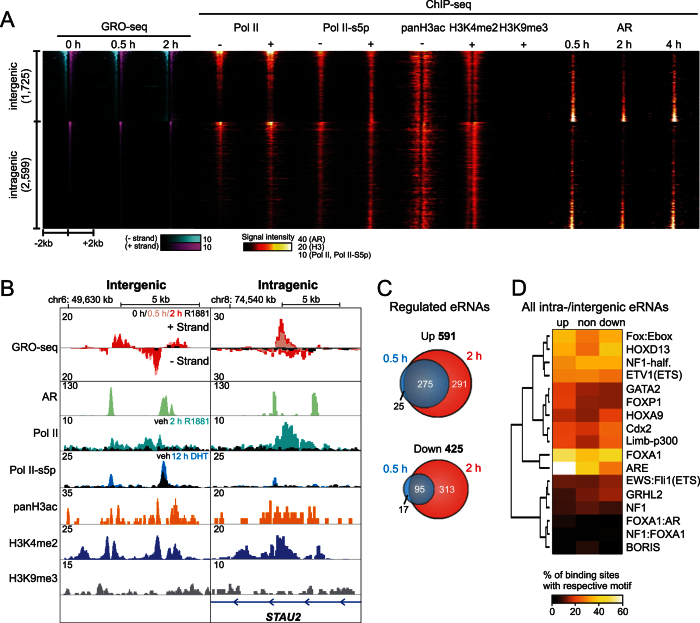
Actively transcribed enhancers in VCaP cells. (**A**) Heat map showing GRO-seq signals at different androgen treatment times (R1881; 0, 0.5, 2 h), and ChIP-seq signals of Pol II, RNA polymerase II serine 5 phosphorylation (Pol II-S5p), H3 histone mark (panH3ac, H3K4me2 and H3K9me3) and AR (R1881; 0.5 h, 2 h, 4 h) with (+) or without (−) androgen treatment at the vicinity of inter- and intragenic enhancers. The enhancers are sorted according to the tag densities in vehicle-treated GRO-seq sample. (**B**) Examples of intergenic and intragenic enhancers. Plus- and minus-strand GRO-seq signals at control (black), and 0.5-h (pink) or 2-h (red) androgen treatment aligned with ChIP-seq tracks of AR (R1881 2 h, green), Pol II in vehicle (black) or androgen treatment (R1881 2 h, cyan), Pol II-S5p in vehicle (black) or androgen treatment (DHT, 12 h, blue), panH3ac (vehicle, orange), H3K4me2 (R1881, 2 h, dark blue) and H3K9me3 (R1881 2 h, gray) are shown. (**C**) Venn diagram showing the number of androgen up- and down-regulated inter- and intragenic enhancers at 0.5-h (blue) and 2-h (red) androgen (R1881) treatment times. (**D**) Enrichment analysis of known DNA-binding motifs within all intergenic and intragenic enhancers. For motif analysis, intergenic and intragenic enhancers were combined. Euclidean clustered heat map of top ten enriched motifs shows the percentage of motif occurrence at androgen treatment up-, down- or non-regulated enhancers.

To analyze the enhancer activity in a more stringent fashion, we used the following criteria to define active enhancers: (i) intergenic enhancers (bidirectional) are regions where two opposing transcripts are within 1 kb of each other^34^ and (ii) intragenic enhancers (unidirectional, to avoid noise from the gene transcription) are transcripts that have the opposite polarity to the transcripts from the protein-coding gene, a maximal length of 500 bp and overlap with H3K4me2 histone mark. By this approach, we identified altogether 4,324 eRNA enhancers (1,725 intergenic and 2,599 intragenic ones) in VCaP cells (Fig. 4A). These enhancers co-occurred with Pol II, Pol II serine 5 phosphorylation (Pol II-S5p) and active histone marks, acetylated H3 (panH3ac) and H3K4me2, and were devoid of inactive histone mark H3K9me3 (Fig. 4A). Heatmap in Fig. 4A arranged according to enhancer transcription in vehicle-treated cells interestingly suggested a negative correlation between the enhancer transcription and the AR ChIP-seq signal, which was confirmed by a more thorough analysis (Supplementary Fig. S4A). Upon androgen treatment, however, the situation was reversed, and the enhancer transcription showed a positive correlation with the AR ChIP-seq signal (Supplementary Fig. S4B). Figure 4B shows examples of transcribed inter- and intragenic sites that, in addition to the AR, are enriched with the Pol II-S5p along with H3K4me2 and H3 acetylation. Nearly one-fourth (23%) of the eRNA-producing enhancers were either induced (~14%) or repressed (~10%) by androgen (log_2_FC ≥ 1 or ≤−1 and FDR ≤ 0.05) (Fig. 4C). Nearly half (45%) of the enhancers that were induced by androgen at 2 h showed induction already at 0.5-h time point, but only about one-fifth (18%) of the enhancers repressed by androgen at 2 h showed repression at 0.5 h (Fig. 4C), suggesting that, as in the case of protein-coding androgen target genes (Fig. 1A), the temporal mode of AR action differs between androgen-induced and -repressed enhancers. However, as the overall co-occurrence of the eRNA-producing enhancers with the all ARBs was merely 22–31%, only a fraction of the ARBs is likely to represent functional androgen-regulated enhancers.

To gain more insight into the TFs that are significant for androgen-regulated enhancers, we performed DNA motif enrichment analysis of androgen up-, down- and non-regulated enhancers (Fig. 4D). A motif for the pioneer factor FOXA1 was abundant in all enhancer categories. Motifs of HOXD13, FOX:Ebox, NF1-halfsite and ETV1 were nearly equally enriched with androgen-induced and androgen-repressed enhancers, whereas motifs of GATA2 and FOXP1 were slightly more enriched among the induced than the repressed enhancers. Notably, the ARE motif was the most unevenly distributed motif between the induced and repressed enhancers; 62% vs. 28%, respectively. This implies that the AR’s action on the androgen-repressed enhancers relies to a clearly lesser extent on direct DNA interaction than its action on the androgen-induced enhancers.

### Holo-AR recruits PIAS1, HDAC3 and ERG to the androgen-induced enhancers

**Figure 5:**
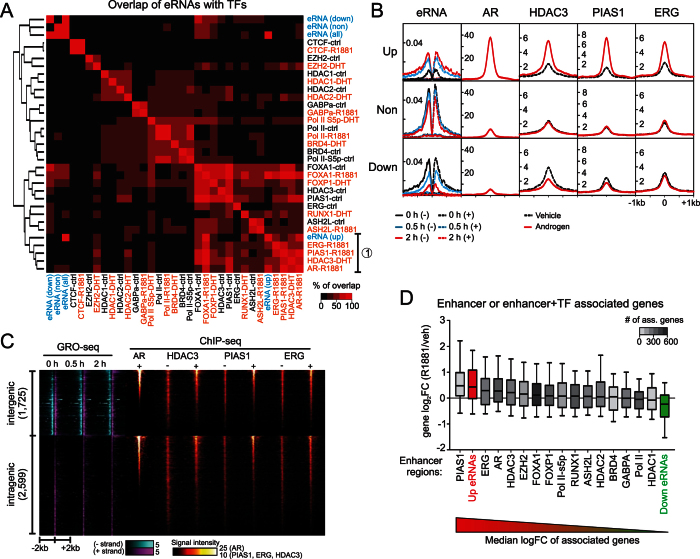
Androgen up-regulated enhancers harbor AR, PIAS1, HDAC3 and ERG. (**A**) Heat map of the co-occurrence of up-, down- and non-regulated enhancers, TFs and transcriptional regulators in VCaP cells. The heat map color indicates the maximal overlap percentage between two data sets (i.e. the percentage of co-occurring sites in the smaller group). GRO-seq defined enhancer datasets are shown in blue, and ChIP-seq binding sites from vehicle (black) or androgen treatment (orange). Cluster of proteins that are most highly associated with up-regulated enhancers is indicated with number one. (**B**) Average tag profiles of GRO-seq and ChIP-seq signals of AR, HDAC3, PIAS1, ERG at up-, down- and non-regulated intergenic enhancers with ±1 kb window. (**C**) Heat map of inter- and intragenic enhancers with tag densities of AR, HDAC3, PIAS1, ERG signals with ±2 kb window. The heat map is sorted according to the tag densities of AR. (**D**) Box-plot showing transcriptional changes of genes associated to androgen-regulated enhancers and TF-associated enhancers. Enhancers were associated with closest TSS of transcribed gene (in 2 h androgen exposure) within ±50 kb. Base 2 logarithmic fold changes (log_2_FC) of the associated transcribed genes were plotted. Associated genes are presented in decreasing order of median log_2_FC in androgen treatment. Whiskers mark 10% and 90% boundaries and line denotes median.

To analyze the role of multi-protein complexes in androgen regulation, we next computed a clustered pair-wise co-occurrence matrix of all TFs or other transcriptional regulators for which VCaP cell-specific ChIP-seq datasets were available^9^,^10^,^13^,^35^,^36^,^37^,^38^. In addition to ChIP-seq data sets from androgen- or vechicle-treated VCaP cells, androgen up-, down- and non-regulated eRNA enhancers were included into analysis (Fig. 5A). The up-regulated enhancers clustered to a completely different group than the down- or non-regulated enhancers. In addition to the AR, the androgen-induced enhancers clustered with PIAS1, HDAC3, and ERG (cluster 1 in Fig. 5A). Because PIAS1, HDAC3 and ERG grouped to different clusters in the absence of androgen, the holo-AR is likely to recruit them to the active enhancers. Interestingly, this analysis showed no evidence for androgen-promoted recruitment of any of the TFs or transcriptional regulators (for which VCaP ChIP-seq data were available) to the androgen-repressed enhancers (Fig. 5A). However, the genome-wide occurrence of FOXA1, HDAC3, ERG, and ASH2L in the absence of androgen showed some overlap with the androgen down-regulated enhancers, suggesting their involvement in enhancer regulation prior to androgen-induced down-regulation. A closer inspection of cluster 1 factors further indicates androgen-induced binding of HDAC3, PIAS1 and ERG to the androgen-induced enhancers (Fig. 5B). Compared to the androgen-induced enhancers, binding of the cluster 1 factors is clearly weaker at androgen down- or non-regulated enhancers in androgen-treated VCaP cells (Fig. 5B). Moreover, androgen attenuated the binding of HDAC3 and PIAS1 at the repressed enhancers which, based on the absence of AR signal at these sites, is independent of the AR binding (Fig. 5B). The androgen-induced binding of cluster 1 factors was also apparent in AR ChIP-seq signal-sorted heat map of eRNA enhancers, further suggesting that holo-AR binding is guiding chromatin binding of these factors in VCaP cells (Fig. 5C).

Next, we analyzed how the genes associated with different enhancer groups respond to androgen. In this analysis, we considered not only up- or down-regulated eRNA enhancers, but also enhancers that co-occur with TFs or co-factors in androgen-treated cells (Fig. 5A). We searched the closest gene TSSs within 50 kb of the enhancer and plotted the androgen-mediated transcriptional changes of these genes. As expected, the analysis revealed that the changes in the enhancer transcription positively correlated with the changes in the gene transcription (i.e. androgen up-regulated enhancers associated preferentially with androgen up-regulated genes; Fig. 5D). The eRNA enhancers co-occurring with PIAS1, ERG and AR associated with genes that showed the most robust induction by androgen (Fig. 5D). Moreover, since the transcriptional changes in the genes associated with PIAS1, ERG and AR were not statistically different from those of genes that associated with up-regulated enhancers, these results suggest that, in addition to the AR, also the PIAS1 and the ERG are involved in AR-mediated gene up-regulation.

We next subjected the genes (416) that associated with the enhancers occupied by PIAS1, ERG and AR to IPA analysis. The analysis showed enrichment of IGF-1 signaling pathway (p-value 4,97E-2) and cholesterol biosynthesis pathways (p-value 2,83E-2), further attesting the biological relevance of the identified enhancers in androgen-mediated regulation.

## Discussion

AR plays an important role in the development of PCa, and changes in androgen signaling are thought to critically contribute to the development of CRPC. However, our understanding of the gene programs that are directly targeted by the AR in CRPC cells is still limited. One of the challenges in deciphering these gene programs is to identify functionally active AR-binding sites from the vast number of AR-binding sites on chromatin. Recent studies have revealed that functionally active enhancer regions are often transcribed and produce short non-coding RNAs called eRNAs^23^,^26^. This enhancer transcription is known to predict transcription of enhancer-associated genes^23^,^26^,^39^. In this work, we used VCaP cells as model CRPC cells to investigate the androgen-directed transcriptional programs by utilizing GRO-seq and also monitoring the changes in the transcription of androgen-regulated eRNAs. In addition, the observed early changes in the transcription were correlated with the cell type matching data of TF chromatin occupancy and histone modifications.

Androgen signaling imposed immediate effects on gene transcription in VCaP cells. Within 0.5 h, approximately five hundred genes were affected, mostly up-regulated by androgen. Pre-establishing an Pol II complex at promoter, or promoter proximal pausing, is one of the ways to quickly induce gene transcription as a response to external stimuli^32^. However, this does not seem to occur with the majority of AR target genes, as they showed lower than average pausing ratios in our analysis. After 2-h androgen stimulus, almost two thousand genes were differentially transcribed, and the difference between up- and down-regulated genes was balanced out. Androgen-induced short-term gene regulation seems to be rare, as the majority of androgen-regulated genes continued to be similarly regulated at 2-h after androgen stimulus. Early gene regulation events of steroid hormone signaling have been studied in also breast cancer cells. Hah *et al*.^40^ observed roughly equal numbers of up- and down-regulated genes after 40-min estrogen induction, whereas after 2-h induction, most (~75%) of the genes were down-regulated. This suggests that, in terms of gene activation or repression and the dynamics of direct gene regulation, androgen action in prostate cancer cells differs from that of estrogen in breast cancer cells.

The androgen-regulated genes in VCaP cells are several-fold outnumbered by the ARBs that mainly localize to intergenic and intragenic regions^9^,^11^,^15^,^17^. Comparison of the AR cistrome from ERG fusion-expressing VCaP cells with those from non-ERG fusion-expressing CaP cell lines suggests that the ERG expression does not modulate the AR cistrome to any large extent. Analyses of enhancer transcripts have shown that intergenic and intragenic enhancer regions typically produce non-coding eRNA transcripts that play a part in gene regulation^23^. These actively transcribed non-coding regions are likely to be engaged with chromatin looping^39^. According to our stringent analyses, only <3% of the intergenic and intragenic ARBs qualified as androgen-regulated eRNA-producing enhancers. These enhancers showed similar androgen responses as androgen-regulated genes, with the majority (>80%) of the transcripts being similarly regulated at both androgen exposure time points. The changes in the enhancer transcription positively correlated with the changes in the nearby gene transcription, which is in line with recent studies in breast cancer cells^20^,^40^, mouse macrophages^23^ and mouse cortical neurons^26^. Interestingly, a group of AR-regulated eRNAs was recently shown to be up-regulated also in CRPC patient specimens^41^. Moreover, long-term enzalutamide, a second generation antiandrogen, treatment was reported to induce alterations in AR-regulated eRNAs, which was suggested to contribute to enzalutamide resistance in CRPC^42^.

**Figure 6:**
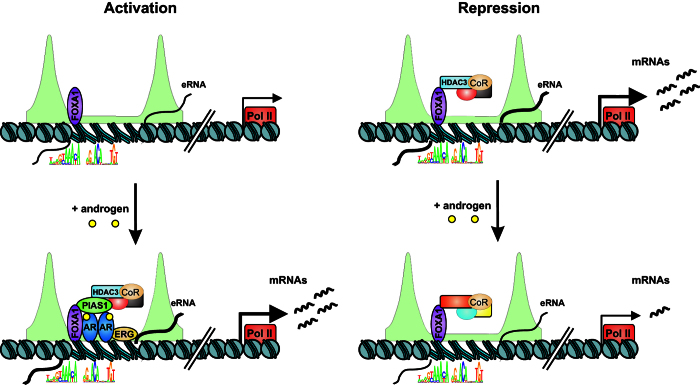
A schematic model of the function of androgen-regulated eRNA-producing enhancers. Androgen-repressed and -activated eRNA-producing enhancers are surrounded by H3K4me2 (line profile filled with green) and pre-primed by FOXA1 (purple) already in the absence of androgen. Holo-AR recruits PIAS1, ERG and HDAC3 and other coregulators (CoR) to the androgen-activated enhancers, whereas the androgen-repressed enhancers do not generally exhibit binding of AR, PIAS1, ERG or HDAC3.

The androgen-regulated enhancers showed differential enrichment of TF-binding motifs. The FOXA1 motif was similarly abundant at up- and down-regulated enhancers, whereas the ARE motif was more than two-fold more abundant at up- than down-regulated enhancers. Also GATA2 and FOXP1 motifs were more enriched at the androgen up- than down-regulated enhancers. These findings imply divergent patterns of TFs in the androgen induction and repression of enhancers. Comparison with the genome-wide maps of VCaP cell TFs indeed showed that androgen up- and down-regulated enhancers differ in their pattern of TFs. In addition to the AR, PIAS1, HDAC3 and ERG were loaded in an androgen-dependent fashion to the androgen-induced enhancers, but not to the androgen repressed enhancers, suggesting that down-regulation of enhancers occurs independently of these TFs, as illustrated in our schematic model in Fig. 6. Interestingly, according to a recent report, ERG in cooperation with HDAC1, -2 and -3 is mediating repression of AR activity on genomic regions that are co-occupied by the receptor and the ERG^10^. These data together with our results suggest that androgen-induced enhancers rapidly recruit repressive factors to the activated enhancers. This could be a mechanism for limiting the androgen signaling on activated enhancers. In line with recent genome-wide studies showing that signal-dependent TFs are not generally present at the enhancers that are rapidly repressed by these signals^43^, androgen seems to repress enhancers through an indirect mechanism, possibly through coregulator squelching^43^.

In conclusion, the function of AR on distal functional enhancers in VCaP CRPC cells involves PIAS1, HDAC3 and ERG that play a role in direct androgen regulation of cellular growth and control signaling pathways. These pathways include IGF-1 and EGF signaling that have been strongly linked to the PCa progression^2^.

## Materials and Methods

### Cell culture and hormones

VCaP cells were obtained from American Type Culture Collection (ATCC, Manassas, VA) and maintained as previously described^44^. For RT-qPCR, GRO-seq and ChIP-seq, VCaP cells were seeded onto 6-well plates (0.5 × 10^6^ cells/well, RT-qPCR), 10 cm dishes (5 × 10^6^ cells/dish ChIP-seq) or 15 cm dishes (8 × 10^6^ cells/dish GRO-seq), grown for 72 h in growth medium and 48 h in steroid-depleted medium (DMEM containing 2.5% charcoal-stripped FBS) prior to R1881 exposure (methyltrienolone, synthetic androgen from Steraloids Inc., Newport, RI, USA).

### RNA isolation, real-time quantitative PCR (RT-qPCR) analysis

VCaP cells in steroid-depleted medium were treated with vehicle (EtOH, 2 h) or R1881 (10 nM) for indicated times. Total RNA was extracted from three biological samples (TriPure isolation reagent, Roche) and converted to cDNA (Transcriptor First Strand cDNA synthesis Kit, Roche) according to the manufacturer’s instructions. Specific primers were used for measurement of target gene expression (*IGF1R*: 5′-AGAAGCCGATGTGTGAGAAG-3′; 5′-TAGTAGTAGTGGCGGCAAGC-3′; *EGFR*: 5′- GTAACAAGCTCACGCAGTTG-3′; 5′-CCTCCTGGATGGTCTTTAAG-3′) by RT-qPCR as described^45^ using *GAPDH* (5′-TGGGGAAGGTGAAGGTCGG-3′; 5′-TCTCAGCCTTGACGGTGCC-3′) mRNA levels to normalize the amount of total RNA between the samples.

### GRO-seq libraries

VCaP cells in steroid-depleted medium were treated with vehicle (EtOH, 2 h) or R1881 for 0.5 h and 2 h. Samples were produced as two biological replicates essentially as described before^39^. Nuclei from ~10 million cells were collected in swelling buffer, run-on reactions were performed in the presence of Br-UTP, RNA was isolated using TRIZOL-LS reagent (Life Technologies), and labeled RNA molecules were affinity purified using agarose-conjugated anti-BrdU (sc32323AC, Santa Cruz Biotechnology). Labeled RNAs were processed for next-generation sequencing with minor modifications from a published protocol^46^. Samples were sequenced with HiSeq 2000 at EMBL genomics core facility (Heidelberg, Germany).

### GRO-seq data analysis

GRO-seq reads were quality controlled using FastQC and FASTX-toolkit (http://hannonlab.cshl.edu/fastx_toolkit/) (minimum 97% of bps over quality cutoff 10). GRO-seq read trimming and mapping against rRNA and human reference genome (hg19) was done as described previously^47^. Gene body RPKM cut-off >0.5 was used to differentiate between transcribed and non-transcribed genes. EdgeR^48^ was used for differential gene expression analysis. Criteria for differentially transcribed genes in androgen-treated versus control sample were RPKM ≥ 0.5, FDR ≤ 0.01 and log_2_FC ≥ 0.585 or log_2_FC ≤ −0.585. Two-hour vehicle sample was used as control sample for 0.5-h and 2-h R1881-treated samples. Heat maps, line profiles and chromatin tracks were done as described previously^47^.

In order to determine transcribed enhancers, GRO-seq libraries (0.5 h R1881, 2 h R1881 and EtOH) were combined to maximize read depth and *de novo* transcripts were identified using HOMER (‘findPeaks’ GRO-seq *de novo* transcript detection)^49^. Transcripts were detected with minor changes to the default setting (transcript initiation sites were detected when GRO-seq read density increased 3-fold relative to the previous 200-bp region and new transcripts were detected when bodyFold change exceeded 3-fold). Enhancers were considered intergenic enhancers, when the enhancer transcript was >3 kb from the gene TTS (to avoid ambiguities caused by transcripts overlapping with annotated transcribed region) and two opposing strand transcripts were identified in maximum 1 kb apart from each other. Intragenic enhancer transcripts were detected from the strand opposite to the underlying gene, overlapped with active enhancer mark H3K4me2 and were short (150 to 500 bp). EdgeR was used for differential enhancer expression analysis. Criteria for differentially regulated enhancers in androgen-treated versus control sample were FDR < 0.01 and log_2_FC ≥ 1 or ≤−1. DNA motif discovery was done using HOMER (‘findMotifsGenome’ using given size for the intergenic enhancers and an area 500 bp upstream of the intragenic enhancers). Pathway enrichment analysis was done using Ingenuity pathway analysis (IPA) to discover enriched pathways and in pathway activity prediction. The canonical pathways with Fisher’s Exact Test p-value < 0.05 and z-score of ≥2 or ≤−2 were predicted to be overall increased or decreased in activity, respectively.

### ChIP-seq data and analysis

ChIP-seq for H3K9me3, CTCF and AR time course samples (0.5 h and 2 h) in VCaP cells was performed as described^45^. Raw reads were quality checked and filtered using FastQC and FASTX-toolkit before alignment of reads against the human genome (hg19) using bowtie 0.12.7^50^, allowing only one mismatch and accepting only the best alignment. Enriched binding sites were defined using findPeaks program of HOMER (software version 4.7.2) with 4-fold enrichment over appropriate background control sample. Peaks found in both biological replicates or all peaks in samples with only one replicate were used in analysis. Binding sites found in both replicates and normalized tag count ≥10 were used as binding sites in the final analysis. In heat maps and line profiles, the ChIP-seq signal was normalized to 10^7^ mapped reads (in samples with replicates the signal was combined and normalized). The ChIP-seq signal comparison analysis, line profiles and chromatin tracks were done as described^47^. The ARBs were associated to the nearest gene promoter in a search window of ±50 kb from ARB to the gene TSS using HOMER.

### Public datasets

Used public datasets in VCaP cells treated with or without androgen: ChIP-seq of AR, PIAS1, FOXA1, Pol II and H3K4me2 (GSE56086)^9^; Pol II-S5p and BRD4 (GSE55062)^35^; panH3ac (GSE56086)^13^; EZH2, HDAC1, HDAC2, HDAC3 (GSE28950)^10^; GABPα and ERG (GSE49091)^36^; FOXP1 and RUNX1 (GSE58428)^51^ and ASH2L (GSE60841)^38^. The peaks detected in Toropainen *et al*.^9^ were used for AR, PIAS1, FOXA1 and Pol II ChIP-seq samples. The ChIP-seq samples in other studies were analyzed as described above without tag normalization.

### Accession numbers

GRO-seq and ChIP-seq data are submitted to the NCBI Gene Expression Omnibus database^52^ (http://www.ncbi.nlm.nih.gov/geo/) and are accessible through GEO Series accession numbers GSE84432.

## Additional Information

**How to cite this article**: Toropainen, S. *et al*. Global analysis of transcription in castration-resistant prostate cancer cells uncovers active enhancers and direct androgen receptor targets. *Sci. Rep.* **6**, 33510; doi: 10.1038/srep33510 (2016).

## Supplementary Material

Supplementary Dataset 1

Supplementary Information
