# Supplementary material for: Global analysis of transcription in castration-resistant prostate cancer cells uncovers active enhancers and direct androgen receptor targets
Source: Sci Rep. 2016 Sep 19;6:33510. doi: 10.1038/srep33510 (PMC5027586; doi:10.1038/srep33510)
Supplement: Supplementary Information [file srep33510-s2.pdf]

Supplementary Material

Global analysis of transcription in castration-resistant prostate cancer cells uncovers active enhancers and direct androgen receptor targets

Sari Toropainen, Einari Niskanen, Marjo Malinen, Päivi Sutinen, Minna Kaikkonen and Jorma J. Palvimo

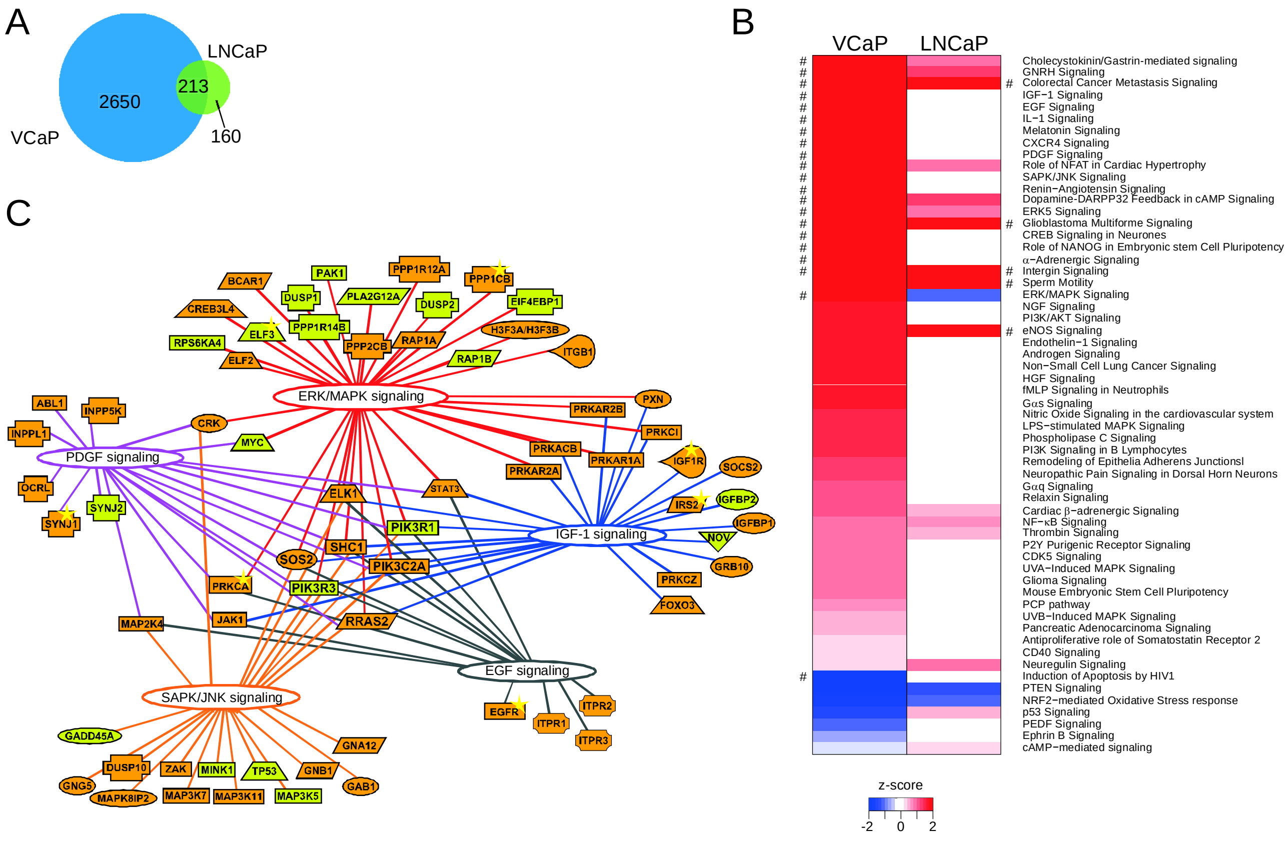

Supplementary Figure S1. Androgen-regulated transcription in VCaP and LNCaP cells. Cells were treated with androgen (5α-dihydrotestosterone, 100 nM) or vehicle for 4 h and subjected to GRO-seq assay. The transcribed genes with RPKM ≥ 0.45, base 2 logarithmic fold change ≥ 0.585 or ≤ -0.585 and FDR <0.01 were considered androgen-regulated genes. (A) Venn diagram showing androgen-regulated genes in VCaP and LNCaP cells. (B) VCaP and LNCaP cells show largely distinct andogen-regulated signaling pathways. Heat map showing canonical pathways predicted to be activated (z-score ≥ 2, red) or attenuated (z-score ≤ -2, blue) in response to androgen treatment in VCaP or LNCaP cells (Fisher's Exact Test p-value <0.05). Significant z-scores are indicated with # mark (z-score >2 or <-2). The color key indicates the activation z-score of the canonical pathway activation (red) or decrease in activity (blue). (C) Interaction map of selected growth and apoptosis pathways in VCaP cells. Genes marked with orange or green background color are up- or down-regulated in VCaP cells, respectively. Yellow star indicates that the gene is similarly regulated by androgen in LNCaP cells.

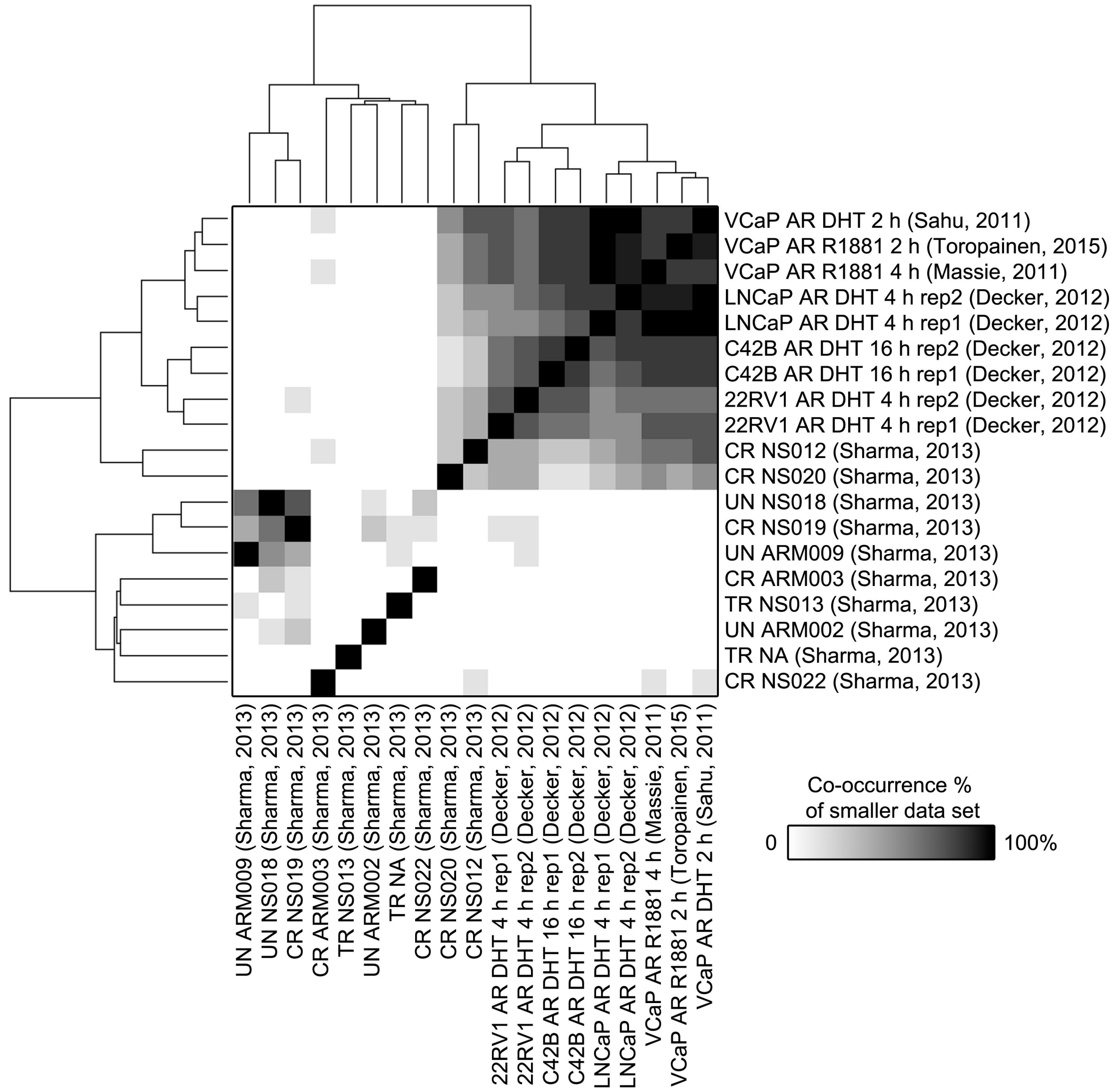

Supplementary Figure S2. Comparison of ARB co-occurrence in prostate-cancer cell lines and clinical samples. Clustering places all ARBs from all cell lines and two castrate resistant (CR) patients in one cluster and two untreated (UN) and one CR in a smaller cluster, while rest of the samples do not belong to any cluster. This suggests that AR binding in cell lines is not largely modulated by the presence of TMPRSS2-ERG fusion that is present in VCaP cells, but not in LNCaP, C4-2B or 22RV1 cells. In addition, ARB pattern in some CR patients resembles that of the cell lines. Co-occurrence of ARBs detected using ChIP-seq were analyzed in HOMER using mergePeaks. The number of ARBs varied between 316 (TR NS013, Sharma et al., 2013) and 41,469 (VCaP Toropainen et al., 2015 GSE56086). Because of the variation in the number of ARBs, overlap percent of the smaller data set was used in the analysis. Overlap percentages were clustered using heatmap.2 in R and are shown in gray scale (0% overlap - white, 100% overlap - black). ARBs were from four cell lines VCaP (Sahu et al., 2011; Massie et al., 2011 GSE28126; Toropainen et al. 2015, GSE56086), LNCaP (Decker et al., 2012, GSE40050), C4-2B (Decker et al., 2012, GSE40050) and 22RV1 (GSE40050), and ten clinical samples from CR, UN and treatment responding (TR) patients (Sharma et al., 2013, GSE28219). Original ARB data were used for all VCaP ARBs, while for other cell lines (LNCaP, C4-2B and 22RV1) raw data were quality controlled, mapped and peaks were called against input sample using settings described in materials and methods. ARBs from clinical samples originally mapped to hg18 (Sharma et al., 2013) were lifted to hg19 using convertCoordinates.pl in HOMER.

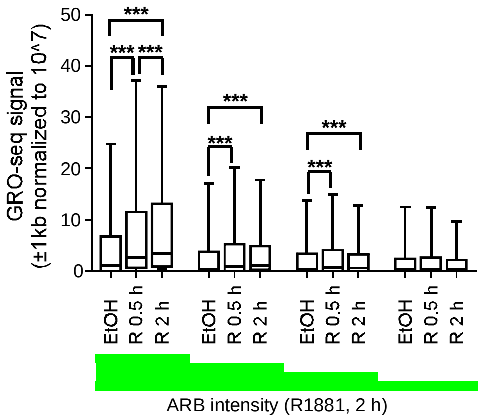

Supplementary Figure S3. Androgen induces transcription at strong ARBs. ARBs were divided into four categories based on the ChIP-seq signal intensity at 2 h R1881 treatment (10 nM, green bar below graph). Transcription changes were analyzed from GRO-seq signal at ±1kb region centered at ARB in vehicle or R1881 (R; 0.5 h or 2 h) treatment. All significant changes within each ARB category are shown. Statistical significances were analyzed in graphpad PRISM (Kruskal-Wallis and Dunn's post test; ***p<0.001). Box edges mark 25% and 75% of data, line denotes the median and whiskers mark 10% and 90% of the data.

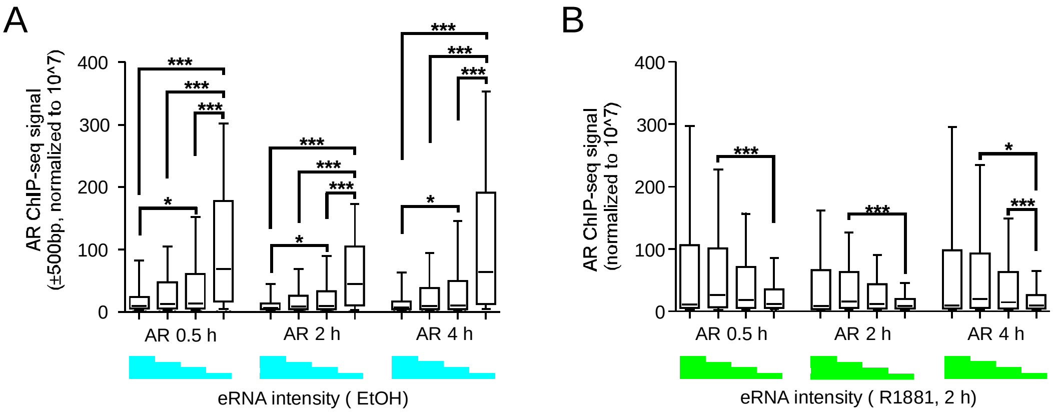

Supplementary Figure S4. Weakly transcribed enhancers co-occur with ARBs that become transcribed upon androgen treatment. Intergenic eRNA enhancers (1,725 enhancers) were divided into four categories based on transcription in vehicle (A; blue) or androgen (B; green, R1881, 2 h) treated cells. Analysis of AR ChIP-seq signal intensities (±500 bp from the center of enhancer, 0.5 h, 2 h or 4 h R1881 treatment) at eRNA enhancer categories revealed a negative correlation of enhancer transcription with AR binding in vehicle treatment (A) but a positive correlation in androgen treatment (B). All significant changes within each ARB category are shown. Statistical significances were analyzed in graphpad PRISM (Kruskal-Wallis and Dunn's post test; *p<0.05, ***p<0.001). Box edges mark 25% and 75% of data, line denotes median and whiskers mark 10% and 90% of the data.
